# Extra Supplement—The Nursing Mirror

**Published:** 1889-06-22

**Authors:** 


### The Nursing Mirror.

All Contributions for this Supplement should be addressed "The Nursing Mirror," care of the Editor, 139-140, Salisbury Court, Fleet Street, London, E.C.

### En Passant.

GRAVESEND HOSPITAL.—At the late annual meeting of governors of this hospital, reference was made to the Nursing Institution established a year ago, which has been most successful. The income has reached £102, the expenditure has been £64, leaving a balance of £37, which will just meet the expense of the district nurse who attends the poor free of charge. The services of the district nurse have not been so eagerly sought as the services of the private nurses; but it is sure to take a little time to accustom the poor to the idea that the nurse will help and comfort them, and not domineer and otherwise upset their modest little households.

TROUBLES AT BIRMINGHAM.—All is not running smoothly at the new workhouse infirmary at Birmingham. The nursing staff has had to be enlarged, and we are glad to say the guardians have found it necessary to engage nurses at £25, as they could not otherwise secure thoroughly competent women. Who the nurses are who accept £16 and £18 a year we have never been able to discover, but it ought to be a rule amongst trained nurses, for their own sake, and for the sake of their fellow workers, never to accept a lower salary than £20. The old nurses at Birmingham receive £20, and they do not see why the new comers should receive a higher wage than they do, hence many are sending in their resignations. Another trouble is that no pauper help is now given; formerly, what might be called the dirty part of ward work was done for the nurse by an able-bodied pauper, but the guardians have now wisely determined to allow no such aid. No nurse ought to grumble at this; a true nurse delights in keeping her ward clean, almost as much as she delights in nursing her patients back to health. It is also better for the whole responsibility of a ward to rest on one pair of shoulders, and we have no sympathy with those nurses at Birmingham who are grumbling because pauper help is denied them.

IN MEMORIAM.—A portrait, executed in oil, of the late Miss Fisher, who, at the time of her death, was matron of the Philadelphia Hospital, was presented on May 28th to the Training School for Nurses connected with the Philadelphia Hospital. The presentation address was made by Senator Hawley, and at the conclusion of the following remarks by Dr. J. William White, senior surgeon to the hospital, the portrait of Miss Fisher, which hung in a conspicuous position, was unveiled: "During the years that Miss Fisher spent here she saw order and neatness and cleanliness, replace disorder and slovenliness and filth. She saw an intelligent and beneficent system evolved from a chaos of ignorance and neglect. She saw hundreds of our most intelligent and charitable citizens visiting and taking an active interest in our institution, of which previously they had scarcely known either the name or location. She saw her pupils going from this hospital to take positions of honour, and to spread her teachings in all parts of the country. She saw the work to which she had consecrated the last years of her life, and to which she had given herself with such an intensity of purpose and self-sacrifice, placed upon an enduring foundation, and realised that here she had an enduring monument. It is not too much to say that her devotion to this work materially shortened her life. Miss Fisher was trained in the Nightingale School, St. Thomas's, London, and before her appointment here was in charge of the hospital at Birmingham."

NOTES FROM NEW YORK.—The graduating exercises of the Mount Sinai Training School for Nurses were held on the 15th May, and those of the Post Graduate School on the 22nd. Mrs. Lewis, the president, in her opening address, made the following remarks: "Training schools for nurses have made it possible for many women to be self-supporting, self-respecting, and generous. The amelioration of the condition of the poor, the improvement made in the nursing service, both of hospital and home, is a splendid result, but not inferior to it is the dignity of possession which they have conferred upon numbers of dependent women. Said a beautiful young graduate the other day, 'How much I do enjoy having my own money!' What was she doing with this money? Spending it on a sick sister! To be able to give one must have; and women have as yet been so poor, they have earned so small a part of the world's wealth! To descend to details: They have raised the price of skilled labour to 3.00dols. per day, and they have decreed that that day shall be but 18 hours long. Is there something yet to be desired? Still we have reason to be thankful. Formerly the nurse's day was 24 hours long." The President of the Post Graduate School, in referring to the death of one of the pupils, who might truly be spoken of as faithful unto death, concluded with these lines:

> Beautiful hands are those that do
> Work that is earnest and brave and true,
> Moment by moment the whole day through.
>
> Beautiful feet are those that go
> On kindly ministry to and fro,
> Down lowliest ways, if God wills it so.
>
> Beautiful shoulders are those that bear
> Ceaseless burdens of homely care,
> With patience, grace, and daily prayer.

A DANIEL COME TO JUDGMENT.—We should not be mortal if we could forbear a little laugh at the *Nursing Record* this week; it has walked into such a comical complication, it will never be able to explain away its own words. This is what it says: "Mr. Ernest Hart, who is generally acknowledged to be a leader amongst English journalists, has for some reason been very cautious in admitting anything but qualified approval of the B.N.A. into the pages of the important paper which he practically has created, and over which he has ruled so long and so well." It then goes on to speak truly of Mr. Hart's "great influence," although all this admiration is excited because the *Record* has jumped at the conclusion that Mr. Hart is going to disapprove of Miss Lückes' pamphlet against registration. But it is rash, even for the most conceited, to rush to conclusions, and bitter indeed must have been the feelings of a certain poor editor when the *British Medical Journal* of Saturday last devoted a column of deserved commendation to this same pamphlet! There was the *Record* proclaiming that Mr. Ernest Hart was *the* leader amongst English journalists, *the* man whose opinion carried weight; there was the *British Medical*, over whose pages Mr. Hart so ably presides, declaring: "In the interests of the nursing world this pamphlet has not appeared an hour too soon. The pamphlet should be read by everyone," etc., etc. And not only praise of the pamphlet but of the writer also, for the notice says that as regards ability, knowledge, and experience, no one has a better right to speak on the subject of registration than Miss Lückes. Poor *Record!* it proclaimed the appearance of a prophet, and lo! the prophet has spoken; and in the utmost amusement we wait to see how the *Record* will receive the judgment it has called down on its own head.

### Whit Monday in a Children's Hospital.

### NORTH COUNTRY CUSTOMS.

Whit Monday in the large Northern towns, besides being a general holiday, is a day specially given up for the children, who look forward to it for weeks before, and, however poor they may be, manage to get some new clothes for the occasion. They assemble according to their parishes, and march with their banners to the principal places in their district, singing a few hymns specially chosen for the day; and then they have a short service in the church, after which they disperse for dinner, to meet again later in the afternoon, when they are taken to some place in the country, where they play games, and after a good tea return home.

Those who are able to be out do not forget their friends in the hospitals, so, as I believe in the South Whit Monday is not made such a children's festival, the readers of The Hospital might like to know how it is spent in the North. The little patients had been busy for some time practising the hymns, for if they could not go with the crowd outside they did not mean to be beaten at the singing. There had been many fears about the weather, because if it had rained the windows could not have been opened, and so half the pleasure would have been gone; but it turned out fine, and every child had on two scarlet jackets to prevent them from catching cold.

The nurses had to be busy very early in the morning to get everything ready, for the first visitors were expected at nine o'clock, and there was a great deal of extra work to be done, one of the standing rules for Whit Monday being that whoever had hair that could possibly be made to curl, it should be curled to the utmost extent. So there were endless papers to be untwisted, and the curls combed out with the greatest elegance. Then those who could be dressed had on the most gorgeous clothes the hospital could produce, and then all the beds had to be turned round with the feet to the windows, so that the occupants could see out; and those who were dressed were perched on tables or high chairs, so that they could see over the heads of the others. Then the finishing touch to the arrangements was to display all the bunting to the greatest advantage. The wards are built in a separate block at the back of the administrative block, the two being connected by a long corridor; this leaves two squares of ground between the two blocks, one on each side of the corridor. Part of one of these squares is a little higher than the ward windows, and it was on this that most of the visitors stood so that they could see into the ward and the patients could see them, all being assembled in one ward for the occasion. Almost before everything was ready the first school came, and there was a great scramble to get all straight, but by the time the school children were marshalled into order—little ones in front, and big ones behind—the wards were quite ready and the windows open. Then the scholars sang to us, and we sang to them, for which they applauded; but that was only a little beginning, as it was a small school. Soon after they had gone the schools of our own parish came. They were accompanied by a great many teachers and friends of the patients, so that every available atom of ground was covered with a dense mass of people, and the conductor had to stand outside one of the windows on the sill. It was a long time before any silence could be procured, the children outside recognising some of the patients, and many being the exclamations at the toys that could be seen in the ward, a fresh army of dolls having been arranged standing on the large locker; but after several futile attempts the clergyman was at last able to make himself heard and announce the number of the hymn. They sang very well and at the tops of their voices, and when they finished they got quite a small storm of applause from the patients, whose turn it was now to sing their hymn. It has been remarked formerly by outsiders that after the roar of so many voices outside the few little voices from the inside sounds most pathetic, but the patients do not think themselves at all to be pittied—in fact, there are generally many old patients who try to get admitted for the time, and if they are fortunate enough to get an invitation for the day they are immensely delighted. The patients got a good round of applause for their hymn, and then the school sang again, after which everyone sang one verse of "God Save the Queen." Then they gave three cheers, or rather an unlimited number, for when once they were started it was no use to try to stop them again. Soon after they went, and another school came, this one numbering about 600 children. They sang remarkably well, and when the patients sang to them they gave them a good round of applause. Also this year for the first time a man went round with a hat making a collection during the singing and brought us the sum of 24s., mostly in coppers, to be spent for the patients.

After waiting a little while and finding no more schools came the patients were refreshed with lemonade, and made tidy for dinner, which is a greater feast than usual.

After dinner a large basket of toys appeared and were distributed. That excitement was hardly over when "The old organ man" arrived. He has been an institution on Whit Monday for the last four years, and, though his organ is almost as old as himself, and quite as wheezy, he is invited to bring it into the ward, and then every child who can by any possibility be moved has the unmixed bliss of grinding it himself. The time is eccentric, for small hands soon get tired, and it sounds very much as if the organ were suffering from an acute attack of St. Vitus dance. The old man has always a great deal to say, but is as deaf as a post. It was discovered this year that he used to recite "pieces" as a boy, so he was called on for one; but he modestly said he thought he should forget, it being forty years since he had done it last, but after a little persuasion he started away with "The Firm Bank," stopping every few lines to explain what it meant. He got triumphantly through the twenty verses, and the children applauded him most vigorously. Being so deaf he did not understand at first, but when he looked round and saw every child clapping as hard as he could he was quite affected, but started off on another piece with renewed vigour. He collapsed after the third verse, owning that "he was pumped out, owing to being always subjected to bronchitis." So he was well supplied with pennies and sent away, being told to be sure to come next year.

Next came tea. It is always the rule for Whit Monday that everyone has buns and coffee for breakfast, but as there is so little time in the morning, everyone being so busy, the patients have theirs for tea. After tea crackers were the order of the day, and then, as Dickens has it, "it was not forty children conducting themselves like one, but each child conducting itself like forty," and it was scarcely possible to get oneself heard above the din of the cracks and exclamations and shouts at the wonderful things found inside. Everything wearable in the shape of hats and capes were immediately put on, one on the top of the other, regardless of style; one little girl with a little pale, patient face adorned herself with a large nightcap and finished off with a fierce black moustache.

The noise now getting rather too uproarious, gingerbreads were distributed, which produced the desired effect of reducing the noise to some extent. Then it was time to go to sleep, a feat which did not take long to accomplish, as all the little patients were thoroughly tired, after having had a most enjoyable day.—Nurse Luke.

### Nurses on their Travels.

### FAREWELL TO MADEIRA.

Funchal, May 30, 1889.

The time has come to leave this lovely climate. Already, beyond the palm and sugar-cane that crown the opposite cliff, I can see the white masts of the "Ceylon," that is to land us in England twelve days from this, calling at Gibraltar and Lisbon on the way. Already, from the endless variety of basket-work, we have selected those most likely to please our friends; also of the pretty dark teal-work, and of embroidery. I had hoped to bring home a living curio, a little sea-horse, who appeared to have a long and happy life (in a French plum jar) before him. Alas, he is dead! In the green weed growing on a stone we had put in for shelter for him lurked some horrid creatures, something between fleas and worms. They fastened on him in the night, and now my poor "Kelpie" is only a preserved specimen with some of those cruel little beasts sticking to him still.

I have visited the Seaman's Hospital. It is a small adapted house, square, white, with green outside shutters, with flattish-tiled roof; the usual style of house here. Inside the rooms are small, dark (not a bad fault in this climate), and to my mind overcrowded with furniture. A sort of radiant cleanliness prevails everywhere, which after the dirt usual in Funchal is positively dazzling. The matron, a Dutch lady trained at Amsterdam, showed me her spotless wards, her homemade horsehair mattresses, her clever and successful fly evictors, her irreproachable medicine and linen cupboards, and her cool blue curtains and hangings. The bathroom, kitchen, and lavatory I did not see, and I gathered from her manner they are not quite so satisfactory. She is a wonderful person, speaks seven languages, has no female assistance, and when I enquired if she did not want a night nurse, declared she would confide her patients to no one—a Sister Dora-like sentiment. I applauded, but with reserve.

Another very interesting institution here is the Orphanage of San Pedro, where there are ten children, but they only occupy a corner of the rambling old palace once belonging to a ducal family, and not without its dark histories. Under its ample roof are sheltered as well a large girls' school, several invalids, and a doctor, who occupies the first floor. The whole is managed, nursed, cooked for, and instructed by a sisterhood, the superior being an Englishwoman, but four nations at least are represented there. Although in the centre of the town the garden is one of the loveliest and certainly the most romantic I have seen. It is reached by a long flight of steps, over-arched and overgrown with flowering creepers, and on the balustrade are chained a small monkey and two grey parrots. I saw the youngest class file down one morning, with their grave dark little faces, and white pinafores, each solemnly kissing the superior's hand as they passed. The garden is walled on one side by a tall stone cliff, across the face of which flows a stream of water in a hidden trench. Below this, trailing mosses, ferns, begonias, and other leafy plants cover all the rock; above all is bare and grey, save on one ledge or terrace, where a mass of arum lilies are rooted, and flourish greatly. A stone tank, lightly roofed over, receives the water, and there all the washing of the establishment is done. The largest camphor tree on the island, one of the largest magnolias, and flowering bamboos shade the place; below is a jungle of lilies, roses, vines, and all imaginable flowers. In this Eden there are, however, not serpents, but pigs! When I exclaimed at this outrage at once on poetry and sanitation, the superior sighed. "They are so profitable, and we are so poor," she said.

Not much longer will this bit of old-world beauty remain; it is required for Government works, and soon sisters, scholars, and orphans must seek a fresh home. Here, as elsewhere, the old giveth place unto the new. There is talk of curtailing the number of processions here, when huge wooden effigies of the saints are carried about the streets accompanied by children dressed as angels. I am glad I have seen Madeira before it is completely modernised.

### Everybody's Opinion.

*[Correspondence on all subjects is invited, but we cannot in any way be responsible for the opinions expressed by our correspondents. No communications can be entertained if the name and address of the correspondent is not given, or unless one side of the paper only be written on.]*

### A NURSES' CLUB.

The Secretary of the Midwives' Institute and Trained Nurses' Club, 15, Buckingham-street, writes: I suppose it is the above club to which your correspondent refers. Any proposal of amalgamation with any other club I should be pleased to lay before my committee, but *our* institute has many other more important objects than that of a social club. We are the only organised body of certificated midwives, and our chief object is to further their compulsory registration. We have also a registry for the convenience of members. I think you would find a club room that was constantly open in any central place a very expensive matter. We, who are entirely self-supporting, find it quite enough to pay a secretary and hire our room once a week. This room is used once a month for medical lectures, and the other evenings as a social meeting place between 5.30 and 10. We have about 15 to 20 members on ordinary nights, who come to look at the medical papers, change books, make enquiries about work, or meet friends. On lecture nights the attendances vary from 30 to 70. Special classes are sometimes held. I think a nurse who wanted a club only would find everything she wanted at the Somerville, Oxford-street, which is open all day and Sundays. Food is easy to procure and cheap. There are papers and a nice library, and most interesting meetings on all sorts of subjects once or twice a week. I think it so much better to join and aid existing institutions that have proved their need, like the Somerville, which has been in existence many years, or our Midwives' Institute (for those nurses who wish for further instruction, etc.), than to multiply new clubs. If we could make our club more convenient to nurses, we should be very pleased to consider any proposal.

### NURSES' EXAMINATIONS.

Nurse Observant writes: The following "medical" questions recently given to first and third years' nurses respectively at one of the leading London hospitals may be of interest to your readers:

First Year's Nurses.

i. Why does a baby cry when a pin is sticking into it?
ii. What are the essentials of a good diet?
iii. How do you make barley water, toast water, and whey?
iv. How do you give a hot-air bath, and what is its action?
v. How do you make turpentine stupe and poppy fomentation?
vi. What is laudanum?

Third Year's Nurses.

i. What difficulties may occur in nursing a case of bronchitis?
ii. What is the best thing for a nurse to do when a child's nose bleeds violently?
iii. Draw out the rules for nursing a case of enteric fever?
iv. Write out the D.C., D.D., and D.L. diets of this hospital, and state to which classes they belong?
v. What are the signs of unconsciousness in a patient?

I have not been a candidate at either of these examinations, but I have attended the course of lectures upon which these questions are supposed to hinge, and I am consequently in a position to express an unbiassed opinion on the subject. There was a time, sir, when women became nurses, and good nurses, too, without being examined at all. I do not advocate a return to such a state of things, nor do I complain of there being two examinations, but I most emphatically contend that the principle of this double examination is the same as that which prompted the London colleges to institute two or more professional tests for students, instead of only one, as was the case fifty years ago. Nurses, like students, are now taught a good smattering of anatomy, physiology, and the collateral sciences, and, like students, it is advisable that they should be tested by an early examination on such subjects as may be considered the ground-work of their professional education. Surely, sir this is a conclusion to which a nurse might legitimately come. How is it borne out by the above papers? Is there any great fundamental difference between them? Is there anything to guide a nurse as to what is, or is not, expected of her in her first examination?

The only question on physiology is the first, "Why does a baby cry when a pin is sticking into it?" I am told, upon good authority, that what the examiner desires in answer to this question is a description of the papillæ and sensitive structure of the skin. If this is the answer desired, I consider the wording of this question misleading, trifling, and incorrect. The true answer to such a question involves, not only the description of the papillæ of the skin, but also of the communication of sensory impulses to the brain of the cerebral changes produced, and of the motor impulses developed, and of the complicated muscular and secretory changes involved in the mechanism of crying. It may be useful to nurses to know that this question being interpreted, means "describe the sensory structures of the skin." Why cannot examiners put their questions in a straightforward way, and not as if they were asking riddles? I shall not be at all surprised if, when I go up for my *viva voce*, this gentleman says, "Now, nurse, tell me what I am thinking of." It will be observed that question ii. of the first year's paper is practically the same as question iv. of the third year's. I have only one other matter to point out. The subjects of the last four questions of the first paper were not so much as touched upon by the examiner in his course of lectures preceding the examination, a course of lectures for attending which we (nurses) are mulcted of ten per cent. of our pitiful wage. The conclusion is obvious. Either we are asked questions in our examinations which are not essential to nursing, or we are not taught things in our lectures which are essential. The latter is the true state of the case. We attend under compulsion, and at our own expense, a course of lectures, in which the most essential points of nursing are omitted, whilst our time is *wasted.* I can use no other word in the consideration of such matters as the "Organ of Hearing in the Lobster," the "Bilharzia Hæmatobia" (I hope I have spelt it right), and the "Vermiform Appendix of the Chimpanzee." I had hoped to name some of the ridiculous questions which nurses are asked at this hospital in their *viva voce* examinations, but I fear I have already been too diffuse. The "higher education of nurses" is much discussed in these days, but let our teachers beware, lest in burdening our minds with what is beyond our "ken," they omit that which is essential, and lest in urging us so much to "observe and to think," we lose sight of our great and primary duty, which is, to obey.

### WOMEN AND THEIR WORK.

Sister Rosa writes: After reading the many accounts and discussions in the newspapers concerning Lady Sandhurst's failure to get into the County Council, I cannot help coming to the conclusion that the words of Rebecca in Sir Walter Scott's "Ivanhoe" are true. "Lady," said Rebecca, "the people of England is a strange race." While we are told on all sides that we women are the great majority of the people, and on that account we might not unreasonably suppose we should have the chief hand in the government of the country, the fact is we are not allowed to have the least finger in the pie, not allowed a seat in Parliament to help to frame the laws, and not even allowed to have a vote in the election of members for that august body. Yet at the same time a woman can be sovereign of the country, and indeed our nation owes a great deal to its female sovereigns. Look how the country has prospered under good Queen Bess, Queen Anne, and her present Gracious Majesty. I wonder what Deborah of old would say if she could see her degenerate daughters of to-day; she, who we are told, not only judged Israel, but who led the army on to victory in the battle-field. Let us be thankful, however, that many ways are now opening up to women of being of use in the world and earning their own living, of which nursing is a most important one, and which I believe has a great future before it; but I don't agree with those nurses who object to ladies of position and of comparative wealth entering our ranks, for at present we are a struggling body, the greater number of us, I should think, scarcely averaging £20 a year of income, therefore the more money and influence we get among us, the sooner we will be able to enlarge our borders and branch out into other lands as well as our own, and where we will in all likelihood be more needed and more successful. When we hear of the pittance some poor women's toil brings them, such as making shirts at 11d. the dozen, and the poor soul I read of lately, who was found sitting up in bed a few days after her confinement making matchboxes at 1d. the gross, it makes one feel it is high time we women were endeavouring to find "fresh fields and pastures new" in the way of work, etc., and high time we fought for our interests and progress.

### THE FOOD QUESTION.

A Late Fever Nurse writes: With reference to the paragraphs on "The Food Question" in your issues of the 4th of May and 1st of June, I beg to say that the statement by the twenty-five nurses of the North-Western Hospital, Haverstock-hill, only tends to show that the state of things exposed by a late nurse has no right to be. The dietary table is the same for all the hospitals of the Metropolitan Asylums Board; it is liberal, and more than any nurse can eat. The variation of the food is left to the discretion and judgment of the steward, and I am convinced it is neither the intention of the managers to have their nurses underfed through insufficient food, nor half poisoned by atrocious cooking, inferior butter or margarine, sour beer, mouldy bread, musty eggs and potatoes an Irishman would be ashamed to give to "his bosom friend," but a short-sighted policy on the part of some of the officials, who, if they do not know the difference between good, wholesome, and decently prepared food, ought to be taught different, and over whom a careful supervision ought to be exercised by the higher authorities. A "Late Nurse" was wrong in stating the nurses complained in a body to the medical superintendent; she should have said a few of the nurses complained. In the hospital referred to, as in other institutions, some there are who are afraid of speaking up, others who cannot afford to complain for fear of losing sight of the longed-for pension which comes as a reward for their long services and abilities. It may be gratifying to A Late Nurse to hear that the House Committee have made an attempt at some enquiry into the matter, and have to all appearance put it down to discontent, and let the matter die a natural death. What the nurses really want is that the managers go into the matter earnestly and see for themselves, and we may expect to know why so many nurses are laid up with dyspepsia, anæmia, etc., and what is done with the ratepayer's money.

### Appointments.

Beckenham Cottage Hospital.—Miss M. J. Costford has been elected matron of this hospital, and will enter upon her duties on July 1. Miss Costford trained at the London Hospital, where she passed a satisfactory examination in 1885. She was then appointed staff nurse, which post she held two years. Since then she has acted as staff nurse in Hope Ward at St. Bartholomew's, and is now being instructed there in housekeeping. Miss Costford has made many friends in the nursing world, who will be glad to hear of her promotion. Matrons especially have always appreciated Miss Costford's nursing powers, and have readily given her those excellent testimonials which have doubtless gained her her present post.

### Vacancies.

The following vacancies are announced:

**Matrons.**

Chichester Infirmary, £50.
Manchester Royal Eye Hospital, £80.

**Sister.**

Bolton Infirmary, £25.

**Nurses.**

Bedford Institute, £26.
Blackheath Institution, £20.
Bradford Infirmary, £25.
Carmarthen Infirmary, £23.
Children's Hospital, Pendlebury.
Dr. Horton, Forest Hill, £20.
East Suffolk Hospital, £20.
Hampshire Nurses' Institute.
Harrogate Cottage Hospital.
Ipswich Hospital, £20.
Kingston Nurses' Home, Hull.
Lowestoft Hospital, £25.
Miss John's Home, Glasgow.
Nurses' Home, Harrogate.
Northamptonshire Nurses' Institution.
Nurses' Institute, Canterbury.
Princess Alice Hospital, Eastbourne, £16.
Potter's Bar Cottage Hospital, £30.
St. George's Home, Sheffield.
Southport Nurses' Home.
St. Cuthbert's Poorhouse, £25.
Surbiton Cottage Hospital.
Wantage Cottage Hospital, £25.
Workhouse Infirmary Nursing Association.

**District Nurses.**

Burton-on-Trent Nursing Institution, £25.
Ballymena (two), £30.
Wantage Cottage Hospital, £25.

**Probationers.**

Bolton Infirmary.
Bushey Heath Convalescent Hospital.
Children's Hospital, Birmingham.
Consumptive Hospital, Ventnor.
Royal Cornwall Infirmary, £9.
St. Helens (Lancs.) Cottage Hospital.
Victoria Infirmary, Northwich.

### Notes and Queries.

**Queries.**

(30) *Hollond Institution.*—"Parish Nurse" would be greatly obliged if through your valuable paper, The Hospital, she could find out if any trained nurses, now in England, have ever nursed in connection with the "Hollond" Institution, Nice (superintendent, Miss Woodcock), and if they consider it a desirable institution for English nurses to join who desire private nursing abroad.

(31) *Free Training in Midwifery.*—Where can it be obtained? *Helen.*

(32) *Droitwich.*—Are the Droitwich baths good for rheumatism, and can cheap lodgings be had there? *A Sufferer.*

(33) *Dispensing.*—Where can I be quickly taught the elements of dispensary work? *M. S. B.*

(34) *Hospitals in France.*—Will you kindly tell me if there is any hospital in France where English nurses who know no French are taken? *F. F.*

(35) What schools for dispensary are there where articled pupils can be received? I want to start a course at once.

**Answers.**

(25) *Pay Patients.*—Try St. John's Hospital, Lewisham. *A correspondent.*—Apply to the Establishment for Invalid Gentlewomen, 90, Harley-street, W. *F. M.*

(31) *Free Training in Midwifery.*—The Workhouse Infirmary Nursing Association, 6, Adam-street, Strand, is now advertising such training. *Editor.*

(32) *Droitwich.*—In The Hospital for November 3rd, 1888, an article appeared on this subject. The baths have a wonderful effect on some cases of rheumatism. Lodgings are not very dear.

(33) *Dispensing.*—Apply at the Zenana Medical College, 58, St. George's-road, S.W., or to Dr. Annie McCall, 131, Clapham-road, or to the New Hospital for Women.

(34) *Hospitals in France.*—Apply to Hertford Hospital, Paris; or to the Levick Institution, 34, Rue de Prony, Paris, where English nurses are taken.

(35) Apply to the Secretary, The Pharmaceutical Society, Bloomsbury-square.

*Nurses as Stewardesses.*—We must again make known the fact that the register of nurses willing to act as stewardesses is closed for the present, and will not be reopened till those now on the list have had their chance of trying a voyage.

